# A Law Regulating the Practice of Dentistry in Kentucky

**Published:** 1878-05

**Authors:** 


### A LAW REGULATING THE PRACTICE OF DENTISTRY IN KENTUCKY.

In answer to the petition of the dental profession of Kentucky, the following law has been enacted by the legislature of that State, and is now in force.

LAW RELATING TO DENTISTS.

*An act to amend an act, entitled "An act to incorporate the State Dental Association," approved February 6, 1868*

SECTION I. *Be it enacted by the General Assembly of the Commonwealth of Kentucky,* That it shall be unlawful for any person to practice dentistry in the State of Kentucky for compensation, unless such person has received a diploma from the Faculty of a Dental College, duly incorporated under the laws of this or any other of the United States or foreign country, or a certificate of qualification issued by the Kentucky State Dental Association: Provided, That nothing in this section shall apply to persons now engaged in the practice of dentistry in this State.

SEC. 2. There shall be a Board of Examiners, to consist of three practitioners of dentistry, who, together with the President and Secretary of the Kentucky Dental Association, shall be elected by said Dental Association, according to its by-laws.

SEC. 3. It shall be the duty of said Board of Examiners, so elected, to meet annually at the time of meeting of said Kentucky Sate Dental Association, or oftener, at the call of any three of the members of said board, or of an applicant for a certificate to practice dentistry.

SEC. 4. Thirty days' notice must be given of the annual meetings of said State Association and previous thereto, that all applicants for certificates to practice dentistry will be granted the same upon satisfactory examination.

SEC. 5. The State Kentucky Dental Association shall cause to be kept a book in which shall be registered the names of all persons having certificates to practice dentistry in the State of Kentucky, and that the book or books so kept shall be a book or books of record, and a transcript from the same, certified by the officer who has it in charge, with the seal of said association affixed thereto, shall be evidence in any court in this Commonwealth.

SEC. 6. Three members of said Board of Examiners shall constitute a quorum for the transaction of business, and should a quorum not be present on the day appointed for their meeting, those present may adjourn from day to day until a quorum is present.

SEC. 7. Any person who shall, in violation of this act, practice dentistry in the State of Kentucky for a fee or reward shall be liable to indictment by the grand jury of the county in which the offense is committed, and, upon conviction, shall be fined in the penal sum of not less than fifty nor more than two hundred dollars for each offense, provided that nothing in this act shall be construed to prevent physicians and surgeons from extracting teeth.

SEC. 8. On the trial of indictments found as aforesaid, it shall be incumbent on the defendent to show that he has authority under the law to practice dentistry, to exempt himself from the penalty by law prescribed.

SEC. 9. All fines collected under this act shall inure to the benefit of common school education, and be added to the fund of such common school in the county in which the offense is committed.

SEC. 10. In order to provide a fund to carry out the provisions of the third section of this act, it shall be the duty of the Board of Examiners to collect from all who receive the certificate to practice dentistry a sum not to exceed twenty dollars each, of which sum, if there be any remaining after paying necessary expenses, the balance shall be paid into the treasury of said Kentucky State Dental Association, to be kept as a fund for the purpose of carrying out more fully and perfectly the provisions of this ac.t

SEC. 11. The Board of Examiners shall receive such remuneration for their services as the by-laws of said Kentucky Dental Association may provide.

SEC. 12. This act shall take effect and be in force from and after its passage.

It will be seen that this law is only prospective in its operation, and will necessarily have the support of every well wisher of the profession in the state.

A faithful execution of its provisions and requirements, will arrest the growth of quackery and empiricism, though it will not remove that which now exists. It will ultimately bring the profession in the state upon a good footing.

The machinery for its execution should at once be put into condition for work,—as it doubtless will.

The State Society should at once procure a correct list and record of all who were in practice at the time of the passage of the law; and then every member of the profession in the state should see to it that the law is obeyed in his own locality. These matters should not be overlooked at the approching meeting of the State Society.

